# Clinical and PET/CT metabolic imaging characteristics across the evolving spectrum of visceral leishmaniasis and associated hemophagocytic lymphohistiocytosis

**DOI:** 10.3389/fimmu.2026.1812725

**Published:** 2026-07-13

**Authors:** Chao Yang, Yan Wang, Ruixian Duan, Feiyan Wei, Xiu Sun

**Affiliations:** Department of Infection, Shanxi Bethune Hospital, Shanxi Academy of Medical Sciences, Third Hospital of Shanxi Medical University, Tongji Shanxi Hospital, Taiyuan, China

**Keywords:** hemophagocytic lymphohistiocytosis, metabolic imaging, positron emission tomography, risk stratification, visceral leishmaniasis

## Abstract

**Objective:**

To delineate the clinical and PET/CT metabolic imaging characteristics across the spectrum of visceral leishmaniasis(VL) and associated hemophagocytic lymphohistiocytosis (HLH), and to develop a simple risk score for identification of patients at risk of visceral leishmaniasis-associated hemophagocytic lymphohistiocytosis (VL-HLH).

**Methods:**

This retrospective study enrolled 21 VL patients categorized into three groups: visceral leishmaniasis only (VL-only, n=7), VL-HLH (n=8), and a clinically indeterminate Grey Zone group (n=6). Clinical and laboratory parameters were compared across groups. For 9 patients with PET/CT, metabolic patterns and correlations between splenic SUVmax and serological markers were analyzed. A simple risk score was constructed based on significant indicators and evaluated across groups.

**Results:**

No differences were observed in age, sex, and main symptoms among the three groups. Compared to the VL-only group, the VL-HLH group exhibited significantly higher levels of the inflammatory marker C-reactive protein and greater spleen thickness (P<0.05). Correlation analysis revealed a multi-indicator interaction network centered on “inflammation-coagulation-tissue injury.” PET/CT identified the spleen (9/9, 100%) and bone marrow (7/9, 77.8%) as the primary metabolic target organs; splenic SUVmax showed strong positive correlations with D-dimer (r=0.82, p<0.01) and ferritin (r=0.69, p<0.05). The simple risk stratification tool (0–2 points) based on C-reactive protein and spleen thickness demonstrated a clear discriminative trend: the VL-only group concentrated at 0 points (85.7%), while the VL-HLH group distributed across 1–2 points. The Jonckheere-Terpstra test for trend confirmed a statistically significant increasing trend in scores with greater disease severity (from VL-only, to Grey Zone, to VL-HLH) (P = 0.002).

**Conclusion:**

This study reveals a systemic “inflammation-coagulation-tissue injury” network in VL, with the spleen as its core metabolic targets. The proposed simple stratification tool based on C-reactive protein and spleen thickness serves as a practical instrument for clinical risk warning.

## 1 Introduction

Visceral leishmaniasis (VL), also known as kala-azar, is a fatal parasitic disease caused by *Leishmania* parasites and transmitted by sandflies. It is classified by the World Health Organization as one of the neglected tropical diseases and represents a significant global public health concern (1–3). An estimated 50,000 to 90,000 new cases occur annually worldwide, primarily in Central Africa, South Asia, South America, and the Mediterranean region (3). In recent years, the main endemic areas in China have been Xinjiang, Gansu, and Sichuan provinces (4). The typical clinical presentation of VL includes prolonged irregular fever, hepatosplenomegaly, and significant weight loss. Definitive diagnosis relies on the microscopic identification of *Leishmania* amastigotes in aspirates from bone marrow, spleen, or lymph nodes, or through specific serological or molecular biological testing (5). In some patients, visceral leishmaniasis progresses to hemophagocytic lymphohistiocytosis (HLH), a life-threatening complication that triggers a vicious cycle of cytokine storm and multi-organ failure, leading to markedly increased mortality (6).

Currently, the diagnosis of visceral leishmaniasis-associated hemophagocytic lymphohistiocytosis (VL-HLH) primarily relies on the internationally recognized HLH-2004 criteria (7).However, as noted in the adult HLH recommendations, these criteria have limitations in adult populations and should be applied with clinical judgment (8). The application of this standard in clinical practice faces two major challenges. First, diagnostic delay. The core laboratory parameters required by the criteria, such as sCD25 and NK cell activity, are often unavailable in most primary healthcare settings, leading to a significant lag in time to diagnosis. This issue is particularly pronounced in resource-limited regions. Even for HLH secondary to common infections like tuberculosis, diagnosis is frequently delayed due to the inaccessibility of these tests (9).More importantly, this “inaccessibility of testing” directly contributes to the second challenge: diagnostic uncertainty. Without access to the key functional assays, a large number of patients can only meet the non-specific items of the HLH-2004 criteria (e.g., fever, cytopenia), thereby falling into a “grey zone” with diagnostic scores of 3–4 points. This state of diagnostic uncertainty is well-acknowledged in HLH research. For instance, the cohort used to develop the widely applied HScore explicitly included a considerable proportion of patients with an “unclear diagnosis” (10). The 2022 Chinese guidelines for the diagnosis and treatment of hemophagocytic lymphohistiocytosis further recognize this diagnostic challenge, stating that patients meeting only 3–4 HLH-2004 criteria should be closely monitored and reassessed (11). Furthermore, the intense inflammatory response inherent to VL itself can produce clinical manifestations highly overlapping with those of HLH (e.g., splenomegaly, hyperferritinemia). It has been explicitly noted in the literature that these non-specific symptoms of VL can mimic HLH, constituting a fundamental challenge for differential diagnosis (12). Consequently, within this “grey zone,” it becomes exceedingly difficult for clinicians to determine whether the symptomatology stems from the infection itself or indicates the superimposition of the life-threatening complication of HLH. In extreme cases, this diagnostic ambiguity can lead to dangerous misjudgment. Some patients with VL, whose clinical presentation fully meets the HLH diagnostic criteria (including pancytopenia, hyperferritinemia, and hemophagocytosis on bone marrow examination), may be misdiagnosed as having “primary” HLH and consequently receive erroneous immunosuppressive therapy (13). Indeed, the literature explicitly cautions that, given the symptomatic similarities and potential co-existence of the two conditions, VL must be promptly ruled out before initiating immunosuppressive therapy for HLH in patients from endemic areas (14). Therefore, systematically elucidating the clinical and biological characteristics along the disease continuum from pure VL to VL-HLH, and exploring simple, reliable indicators—independent of complex functional assays—for early risk stratification, is of urgent clinical significance to overcome the current diagnostic dilemmas.

As an advanced molecular imaging technique, positron emission tomography/computed tomography (PET/CT) enables non-invasive and quantitative assessment of systemic inflammatory metabolic activity. Recent studies have demonstrated that in Epstein-Barr virus (EBV)-associated HLH, quantitative metabolic parameters from PET/CT, such as the maximum standardized uptake value (SUVmax) in the spleen and bone marrow, not only aid in differential diagnosis but also serve as independent predictors of prognosis (15). This suggests that PET/CT may offer a unique perspective for understanding the systemic inflammatory burden in HLH. However, the systematic characteristics of PET/CT in VL and its associated HLH remain largely undefined.

Therefore, this study aims to achieve the following objectives through a preliminary exploratory analysis: first, to systematically delineate and compare the clinical and laboratory profiles of patients with visceral leishmaniasis only (VL-only), visceral leishmaniasis complicated by HLH (VL-HLH), and those in the diagnostic “grey zone”; second, to clarify the metabolic distribution patterns of PET/CT in VL patients and their intrinsic associations with key serological markers. Based on these findings, we anticipate preliminarily outlining a potential stratification tool based on readily available indicators that can suggest the risk of HLH. This will provide crucial hypothetical evidence and directional guidance for the future construction and validation of a true risk-warning tool in large-scale prospective cohorts.

## 2 Materials and methods

### 2.1 Study subjects, sample size, and grouping criteria

This study was approved by the Ethics Committee of Shanxi Bethune Hospital (approval No. YXLL-2026-027). Written informed consent was obtained from all patients. The study was conducted in accordance with the Declaration of Helsinki. This was a single-center, retrospective, observational study. A total of 21 adult (≥18 years) patients with VL hospitalized in our center between August 2022 and July 2025 were ultimately enrolled. The diagnosis of VL required meeting at least one of the following criteria (1): microscopic identification of *Leishmania* amastigotes in tissue smears (e.g., bone marrow); (2) positivity on specific serological tests for *Leishmania* (e.g., rK39); or (3) detection of *Leishmania*-specific nucleic acid sequences in clinical samples via polymerase chain reaction (PCR) or metagenomic next-generation sequencing (mNGS)/targeted next-generation sequencing (tNGS). Exclusion criteria included: pregnancy or lactation; patients with other confirmed etiologies capable of inducing HLH (e.g., active malignancy, autoimmune disease, tuberculosis); and individuals with critically incomplete key clinical data. However, no patients met these exclusion criteria, and all 21 enrolled patients were included in the final analysis.

Among patients meeting the above criteria, grouping was performed based on the HLH-2004 diagnostic criteria and data available from medical records: (1) VL-HLH group: definitively meeting ≥5 items of the HLH-2004 criteria; (2) VL-only group: definitively not meeting ≥5 items and having a mild clinical course; (3) Grey Zone group: meeting 3 to 4 items, or having a highly suggestive clinical presentation but remaining unclassifiable into either of the above groups due to the absence of key diagnostic parameters (e.g., sCD25, NK cell activity).

### 2.2 Data collection

Data were retrospectively collected from the hospital’s electronic medical records, laboratory information, and picture archiving and communication systems. All routine laboratory parameters (e.g., white blood cell count, hemoglobin, platelet count, C-reactive protein, ferritin, coagulation profiles) and ultrasonographic measurements (spleen size, liver size) reported in this study represent baseline values at admission. The highest body temperature recorded during hospitalization is reported. HLH-specific diagnostic markers (e.g., soluble CD25, NK cell activity) were measured when clinically indicated during hospitalization. The collected data included: (1) demographics, symptoms, and signs (age, sex, highest body temperature, presence of fever, bleeding, rash, etc.); (2) laboratory parameters (white blood cell count, hemoglobin, platelet count, C−reactive protein, erythrocyte sedimentation rate, ferritin, and coagulation profiles); (3) ultrasonographic measurements: maximum oblique diameter of the right hepatic lobe, anatomical longitudinal diameter of the spleen, and splenic thickness. Measurements were performed using a Philips EPIQ7 color Doppler ultrasound diagnostic system. For splenic thickness, the oblique intercostal view was used to clearly visualize the splenic hilum and splenic vein along the long-axis plane of the spleen, and splenic thickness was defined as the distance from the splenic hilum to the splenic diaphragmatic surface. All measurements were performed by the same senior physician.(4) comorbidities (type 2 diabetes, hypertension); (5) for the 9 patients who underwent PET/CT, the maximum standardized uptake value (SUVmax) of the spleen, liver, and bone marrow was recorded.

### 2.3 Statistical analysis

Statistical analyses were conducted using R software (version 4.5.1). Continuous variables were presented as median (interquartile range) [M (IQR)], with intergroup comparisons performed using the Kruskal-Wallis test. Categorical variables were described as frequency (percentage) and compared using Fisher’s exact test. Spearman correlation analysis was employed to assess associations between indicators. Receiver operating characteristic (ROC) curves were used to evaluate the discriminative ability of candidate indicators, and the area under the curve (AUC) was calculated. Unsupervised k-means clustering analysis was then performed, followed by variable elimination analysis to assess the importance of each parameter. A risk scoring system was constructed based on indicators that showed good discriminative ability in ROC analysis and were validated by clustering. To further validate the stability of the scoring system, leave-one-out cross-validation (LOOCV) was performed. A 0–2 point scoring system was constructed based on clinical thresholds, and the Jonckheere-Terpstra test was used to analyze the trend of risk scores across groups. All tests were two-sided, and a P value < 0.05 was considered statistically significant.

### 2.4 PET-CT imaging and analysis

PET/CT scans were performed as part of the diagnostic workup for fever of unknown origin, prior to a definitive diagnosis being established. Ultimately, some patients were confirmed to have visceral leishmaniasis by subsequent testing. After fasting for more than 6 hours, patients received an intravenous injection of ¹^8^F-fluorodeoxyglucose (¹^8^F-FDG) at a dose of 4.44 MBq/kg, followed by a 60-minute rest period before whole-body PET/CT scanning. All images were independently reviewed by two nuclear medicine physicians. The maximum standardized uptake value (SUVmax) of the spleen, liver, and bone marrow was measured using the region of interest (ROI) method, and the average value was used for analysis.

## 3 Results

### 3.1 Baseline characteristics of the study population

This study retrospectively analyzed the clinical data of 21 patients with VL admitted between August 2022 and July 2025. According to pre-defined diagnostic criteria, patients were categorized into three groups: VL-only (n=7), VL-HLH (n=8), and a clinically indeterminate Grey Zone group (n=6). The specific HLH-2004 criteria met by the 6 patients in the Grey Zone group are summarized in **Supplementary Table 1**. No statistically significant differences were observed among the three groups regarding age, sex, main clinical symptoms (fever, bleeding, rash), or highest body temperature (all P > 0.05) (**Table 1**). In terms of hematological parameters, the white blood cell count showed the lowest trend in the VL-HLH group [1.60 (1.35, 2.30) ×10^9^/L], with a borderline significant difference across groups (P = 0.051). Platelet count and hemoglobin also exhibited decreasing trends in the VL-HLH group, though these did not reach statistical significance (P = 0.098 and P = 0.078, respectively). The systemic inflammatory marker C-reactive protein (CRP) showed a statistically significant difference in distribution among the three groups (P = 0.028), with levels significantly higher in the VL-HLH group compared to the VL-only group. Coagulation function indicators, including prothrombin time and D-dimer, also demonstrated significant differences across groups (P = 0.041 and P = 0.048, respectively). For both indicators, the VL-HLH and Grey Zone groups had higher levels than the VL-only group; however, *post-hoc* pairwise comparisons indicated that these intergroup differences did not reach statistical significance (all P > 0.05). A significant difference in spleen thickness was observed among groups on ultrasonographic measurement (P = 0.040), with the VL-HLH group having a significantly greater thickness than the VL-only group (**Table 1**). However, no statistically significant differences were observed for several other indicators across the three groups. Serum ferritin, lactate dehydrogenase, liver function indices, renal function, blood lipid profiles, immunoglobulin levels (IgG, IgM, IgA), and the prevalence of common comorbidities (type 2 diabetes, hypertension) were all similar (all P > 0.05). Four of the 21 patients had received corticosteroids before admission (three short-term for fever, one long-term). Routine infection screening revealed no other definite infectious etiologies except for one patient (Grey Zone group) who had concurrent pneumonia.

**Table 1:** Comparison of baseline characteristics among patients with visceral leishmaniasis only, grey zone, and visceral leishmaniasis-associated hemophagocytic lymphohistiocytosis.

Characteristics	All (n=21)	VL-only (n=7)	Grey zone (n=6)	VL-HLH (n=8)	P value^†^
Demographic data					
Age (years)	58.00 (54.00, 69.00)	64.00 (54.00, 70.00)	58.50 (34.00, 60.00)	56.00 (47.00, 68.00)	0.576
Gender (M/F)	17/4	6/1	4/2	7/1	0.636
Symptom					
Fever (n%)	20 (95.2%)	6 (85.7%)	6 (100.0%)	8 (100.0%)	0.619
Bleed (n%)	2 (9.5%)	0 (0.0%)	1 (16.7%)	1 (12.5%)	0.745
Rash (n%)	2 (9.5%)	1 (14.3%)	0 (0.0%)	1 (12.5%)	1.0
Tmax (°C)	39.90 (39.00, 40.00)	38.80 (38.50, 40.10)	39.95 (39.50, 40.00)	39.95 (39.45, 40.25)	0.406
Laboratory indicators					
WBC (×10^9^/L)	2.10 (1.50, 2.50)	2.60 (1.90, 2.90)	1.85 (1.30, 2.20)	1.60 (1.35, 2.30)	0.051
RBC (×10¹²/L)	3.47 (3.14, 3.70)	3.71 (3.14, 3.89)	3.42 (3.14, 3.66)	3.19 (2.73, 3.53)	0.266
HGB (g/L)	94.00 (76.00, 106.00)	106.00 (98.00, 114.00)	88.50 (82.00, 94.00)	85.00 (71.50, 99.00)	0.078
PLT (×10^9^/L)	58.00 (46.00, 92.00)	82.00 (58.00, 181.00)	62.00 (29.00, 109.00)	47.00 (35.00, 55.50)	0.098
CRP (mg/L)	86.00 (33.26, 101.71)	33.26 (14.88, 79.66)^a^	81.40 (30.73, 141.25)	99.52 (90.54, 154.17)^a^	0.028
ESR (mm/h)	38.00 (23.00, 65.00)	32.00 (20.00, 47.00)	55.00 (26.00, 82.00)	42.00 (24.00, 103.00)	0.425
Ferr (ng/L)	1,888.00 (794.00, 3,857.00)	1,806.00 (92.70, 7,663.00)	1,732.00 (964.60, 2,242.00)	3,037.00 (977.00, 7,527.00)	0.543
PT (s)	13.80 (11.90, 14.10)	11.90 (11.50, 12.90)	13.95 (13.90, 16.00)	13.95 (13.00, 14.75)	0.041
APTT (s)	34.20 (31.60, 37.40)	33.50 (30.60, 38.70)	32.50 (29.90, 34.40)	35.05 (32.90, 38.85)	0.299
FIB (g/L)	2.76 (2.22, 3.24)	2.76 (1.98, 3.18)	2.38 (2.22, 3.00)	3.17 (2.26, 3.39)	0.460
D-dimer (ng/mL)	6,752.00 (3,286.00, 11,345.00)	2,702.00 (340.00, 3,318.00)	7,072.50 (5,375.00, 14,257.00)	10,590.50 (5,201.50, 14,258.00)	0.048
LDH (IU/L)	507.50 (227.15, 694.25)	414.40 (196.50, 545.30)	600.25 (252.80, 943.10)	535.10 (258.25, 681.15)	0.763
ALT (IU/L)	33.20 (23.90, 55.30)	39.90 (9.80, 55.30)	51.60 (29.40, 95.70)	30.50 (24.45, 41.85)	0.504
AST (IU/L)	52.50 (34.20, 81.30)	46.70 (15.60, 106.70)	50.75 (25.80, 81.30)	57.20 (36.85, 70.45)	0.950
ALP (IU/L)	76.50 (57.60, 110.80)	88.10 (66.70, 117.20)	68.45 (53.00, 110.80)	58.10 (53.80, 128.05)	0.289
GGT (IU/L)	76.10 (30.60, 115.20)	94.40 (33.00, 126.70)	66.45 (30.60, 104.10)	66.40 (23.15, 122.75)	0.859
ALB (g/L)	27.80 (24.80, 31.90)	32.20 (26.40, 34.10)	28.70 (24.80, 31.90)	26.70 (23.65, 28.55)	0.165
TBil (μmol/L)	12.35 (9.25, 14.80)	14.30 (11.80, 19.10)	11.75 (9.80, 13.00)	11.30 (7.90, 13.90)	0.311
DBil (μmol/L)	3.65 (2.45, 4.75)	4.15 (2.60, 6.20)	3.50 (2.60, 4.60)	3.65 (2.10, 4.65)	0.715
Cr (μmol/L)	83.40 (76.90, 93.10)	80.00 (76.90, 96.30)	82.15 (70.80, 89.10)	90.65 (70.65, 95.15)	0.809
BUN (mmol/L)	5.80 (4.10, 7.30)	4.60 (4.20, 9.30)	6.00 (3.80, 7.00)	5.85 (3.50, 7.65)	0.928
TC (mmol/L)	2.82 (2.36, 3.28)	2.96 (2.09, 3.55)	2.79 (2.36, 3.08)	2.80 (2.47, 3.32)	0.973
TG (mmol/L)	1.59 (1.17, 2.18)	1.73 (0.99, 2.22)	1.77 (1.03, 2.24)	1.53 (1.44, 1.85)	0.940
Immunoglobulin					
IgG (g/L)	20.53 (15.56, 31.16)	17.08 (15.19, 20.15)	25.01 (18.91, 30.17)	22.22 (15.48, 36.86)	0.416
IgM (g/L)	1.14 (0.74, 1.70)	0.93 (0.63, 1.25)	1.61 (1.15, 10.45)	0.90 (0.72, 1.47)	0.067
IgA (g/L)	1.88 (1.57, 2.51)	3.71 (1.97, 4.74)	1.87 (1.81, 2.16)	1.66 (1.49, 1.98)	0.110
Color Doppler US					
Liver (cm)*	13.60 (12.80, 14.40)	14.10 (12.80, 14.40)	12.80 (12.60, 13.10)	13.80 (13.10, 15.10)	0.339
Spleen long (cm)	16.30 (14.80, 18.80)	15.30 (14.00, 16.00)	17.80 (15.40, 19.00)	18.15 (16.30, 19.55)	0.074
Spleen thickness (cm)	6.21 (5.52, 6.85)	5.52 (5.00, 6.19)^a^	6.50 (5.78, 6.92)	6.71 (6.00, 7.20)^a^	0.040
Comorbidity					
T2DM (n%)	4 (19.0%)	2 (28.6%)	1 (16.7%)	1 (12.5%)	0.810
Hypertension (n%)	9 (42.9%)	4 (57.1%)	2 (33.3%)	3 (37.5%)	0.748

Data are presented as median (interquartile range, Q1–Q3) for continuous variables and as number (%) for categorical variables. ^†^The P values shown in the table are for overall comparisons among the three groups (Kruskal-Wallis test for continuous variables; Fisher’s exact test for categorical variables. Values sharing the same superscript letter indicate a statistically significant difference between the two groups.

Liver (cm)*, Liver measurements refer to the maximum oblique diameter of the right hepatic lobe.

WBC, White blood cell; RBC, Red blood cell; HGB, Hemoglobin; PLT, Platelet; CRP, C reactive protein; PCT, Procalcitonin; ESR, Erythrocyte sedimentation rate; Ferr, Ferritin; PT, Prothrombin time; APTT, Activated partial thromboplastin time; FIB, Fibrinogen; LDH, Lactic dehydrogenase; ALT, Alanine aminotransferase; AST, Aspartate aminotransferase; ALP, Alkaline phosphatase; GGT, Gamma-glutamyl transferase; ALB, Albumin; TBil, Total bilirubin; DBil, Direct bilirubin; Cr, Creatinine; BUN, Blood urea nitrogen; TC, Total cholesterol; TG, Triglyceride; IgA, Immunoglobulin A; IgG, Immunoglobulin G; IgM, Immunoglobulin M; T_2_DM, Type 2 Diabetes Mellitus.

### 3.2 Correlation analysis of key clinical indicators

**Figure 1:**
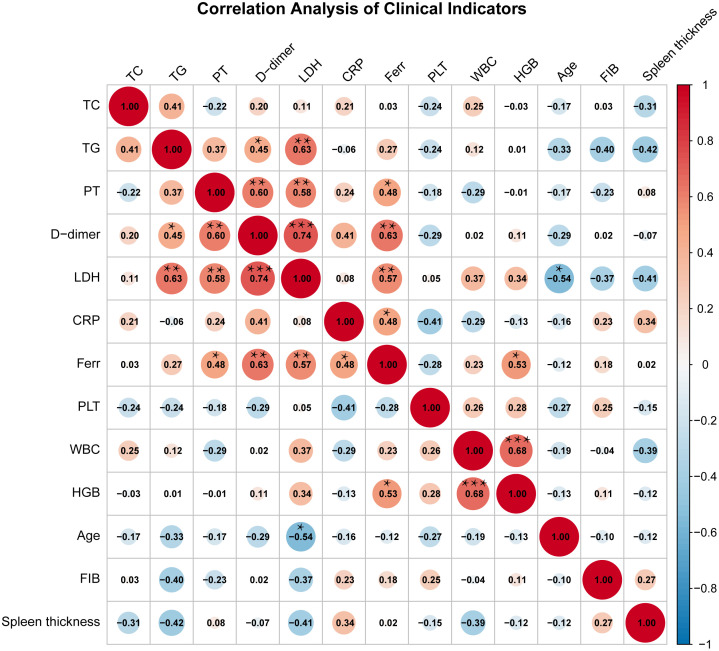
Correlation heatmap of clinical parameters in patients with visceral leishmaniasis. Pairwise correlations among baseline clinical parameters in 21 patients with visceral leishmaniasis were assessed using Spearman’s rank correlation coefficient. Red and blue indicate positive and negative correlations, respectively. Larger bubble size and deeper color represent stronger correlations. Statistically significant correlations are indicated with asterisks as follows: *p < 0.05, **p < 0.01, ***p < 0.001.

To gain a deeper understanding of the coordinated changes among the above-mentioned key indicators (such as CRP, spleen thickness, D-dimer, etc.) during the disease process, we further evaluated the pairwise correlations of these clinical indicators across all patients. Spearman correlation analysis (**Figure 1**) revealed significant associations among multiple clinical indicators. A total of 8 strong positive correlations (r > 0.5) were identified, ranked by strength as follows: D-dimer and lactate dehydrogenase (LDH) (r = 0.74, p=0.0002), white blood cell count (WBC) and hemoglobin (HGB) (r = 0.68, p=0.0007), ferritin and D-dimer (r = 0.63, p=0.0022), triglyceride (TG) and LDH (r = 0.63, p=0.003), prothrombin time (PT) and D-dimer (r = 0.60, p=0.0037), LDH and PT (r = 0.58, p=0.0071), ferritin and LDH (r = 0.57, p=0.0081), and ferritin and HGB (r = 0.53, p=0.0132). One strong negative correlation (r < -0.5) was found: age and LDH (r = -0.54, p=0.013). These association patterns collectively depict a complex pathophysiology in the context of visceral leishmaniasis, characterized by the intricate interplay among the inflammatory, coagulation, tissue injury, metabolic, and hematopoietic systems.

### 3.3 Analysis of PET/CT metabolic imaging features

**Figure 2:**
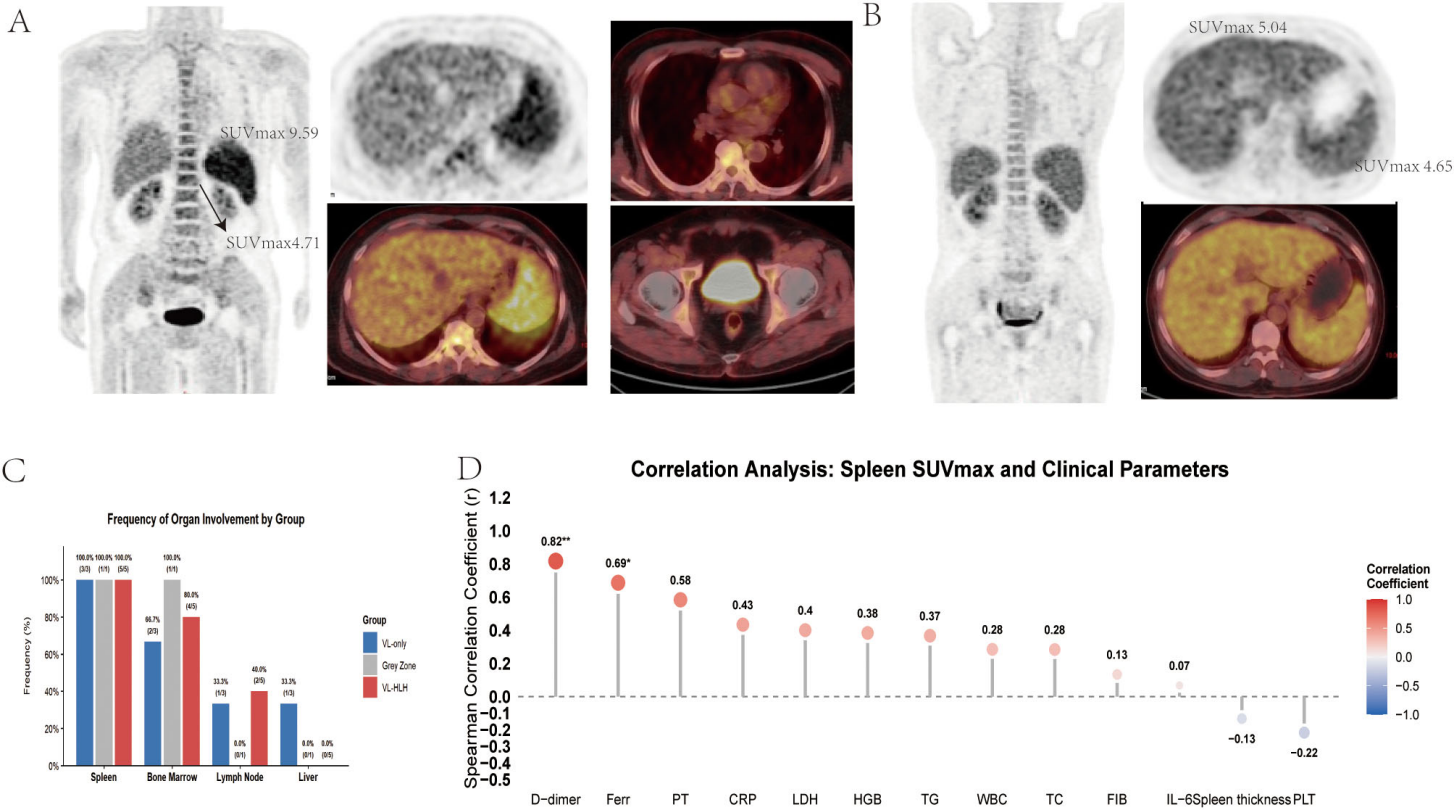
PET/CT metabolic characteristics of visceral leishmaniasis and their correlation with clinical parameters. **(A)** Representative PET/CT image of a patient with VL-HLH; **(B)** Representative PET/CT image of a patient with VL-only; **(C)** Frequency distribution of metabolically active organ involvement by group (n=9); **(D)** Dot plot depicting correlations between splenic metabolic activity (SUVmax) and key clinical parameters. Dots above the baseline indicate positive correlations, those below indicate negative correlations; *p < 0.05, **p < 0.01.

To investigate the manifestation of the aforementioned serological correlations at the organ metabolic level, we analyzed the PET/CT data of 9 patients. All PET/CT scans were performed prior to anti-leishmanial therapy, during the active febrile phase, as part of the diagnostic workup for fever of unknown origin. PET/CT imaging revealed characteristic metabolic distribution patterns. Representative cases visually contrasted the metabolic differences between distinct disease states (**Figures 2A, B**). A patient with VL-HLH exhibited marked splenomegaly with intense hypermetabolism (SUVmax = 9.59), accompanied by diffusely increased metabolic activity in multiple skeletal sites, including the femoral and spinal bone marrow (SUVmax = 4.71) (**Figure 2A**), suggesting a pattern often associated with systemic hyperinflammation. In contrast, a patient with VL-only showed metabolic elevation prominently involving both the spleen (SUVmax = 4.65) and liver (SUVmax = 5.04) (**Figure 2B**), in the absence of definitive bone marrow involvement, indicating a more localized, hepatosplenic-predominant metabolic pattern. Integration of PET/CT data from all 9 patients, stratified by clinical group, revealed that the spleen was the only organ with 100% involvement across all three groups (VL-only: 3/3; Grey Zone: 1/1; VL-HLH: 5/5). For bone marrow involvement, it was observed in 2/3 (66.7%) of VL-only patients, 1/1 (100%) of the Grey Zone patient, and 4/5 (80%) of VL-HLH patients. Lymph node involvement was present in 1/3 (33.3%) of VL-only patients and 2/5 (40%) of VL-HLH patients, with no involvement in the Grey Zone patient. Liver involvement was only observed in one VL-only patient (1/3, 33.3%) (**Figure 2C**). Notably, among 3 patients with clinically documented hepatomegaly, only 1 (11.1%) demonstrated unequivocally increased metabolic activity (elevated SUV) on imaging. Detailed quantitative data (maximum standardized uptake value, SUVmax) for key metabolic target organs in each case, along with group assignment, are presented in **Table 2**. In the VL-only group (n=3), all patients showed splenic involvement. Two of them (2/3, 66.7%) had definitively increased bone marrow metabolism (with SUVmax values of 5.50 and 6.14, respectively). One patient (1/3, 33.3%) presented with cervical lymph node involvement, and another (1/3, 33.3%) had hepatic involvement. In the VL-HLH group (n=5), splenic involvement was similarly universal. Four patients (4/5, 80%) exhibited increased bone marrow metabolism, among whom three had definitive SUVmax values (range: 2.50–4.71) and one was described as “slightly increased”. Concurrently, two patients in this group (2/5, 40%) showed definite lymph node involvement (paraaortic and mediastinal). In Case 06 (Grey Zone), the splenic metabolism was extremely high (SUVmax 9.85) and the bone marrow was described as “increased”, indicating a metabolic pattern more closely aligned with the VL-HLH group. Serum IL-6 levels, measured in the same 9 patients, are also presented in **Table 2**. In the VL-only group (n=3), IL-6 levels ranged from 22.50 to 68.37 pg/mL (median 35.44 pg/mL). In the VL-HLH group (n=5), IL-6 levels showed substantial inter-individual variability (range 1.05-167.40 pg/mL; median 23.30 pg/mL), with no clear increasing trend observed relative to disease severity, likely due to the small sample size and marked individual variability. The single Grey Zone patient exhibited a markedly elevated IL-6 level (221.30 pg/mL), consistent with the hyperinflammatory pattern observed on PET-CT.

**Table 2:** PET/CT metabolic activity (SUVmax) and serum IL-6 levels in individual patients.

ID	Group	Spleen SUVmax	Bone and bone marrow SUVmax	Liver SUVmax	Lymph node SUVmax	IL-6(pg/ml)
01	VL-only	4.65	–	5.04	–	68.37
02	VL-only	8.00	5.50	–	–	22.5
03	VL-HLH	4.44	2.50	–	–	23.3
04	VL-HLH	5.19	Slightly increased	–	–	167.4
05	VL-only	5.26	6.14	–	6.69^a^	35.44
06	Grey Zone	9.85	Increased	–	–	221.3
07	VL-HLH	9.59	4.71	–	–	1.7
08	VL-HLH	5.59	–	–	3.01^b^	1.05
09	VL-HLH	9.56	4.15	–	5.37^c^	108.609

-No evidence of involvement; ^a^Involvement of cervical lymph nodes; ^b^Involvement of para-aortic lymph nodes; ^c^Involvement of mediastinal lymph nodes.

Based on the defined characteristics of involved organ distribution, this study further analyzed the correlation between the metabolic activity (SUVmax) of the core target organ, the spleen, and clinical indicators. The results showed that splenic SUVmax was significantly positively correlated with D-dimer (r = 0.82, p < 0.01), which reflects coagulation function, and with ferritin (r = 0.69, p < 0.05), which reflects iron metabolism and acute-phase response (**Figure 2D**). In addition, SUVmax generally exhibited a positive association trend with other indicators. Among them, the correlations with prothrombin time (PT, r = 0.58), inflammatory markers (CRP, r = 0.43), and tissue damage markers (LDH, r = 0.40) were relatively high but did not reach statistical significance. In contrast, weaker correlations (|r| < 0.4) were observed with routine blood parameters (such as HGB, WBC, PLT), blood lipids (TG, TC), and spleen thickness (**Figure 2D**). In summary, these data show that splenic metabolic activity shows strong positive correlations with key serological indicators of both coagulation dysfunction and systemic inflammation/acute-phase response, implying a tight pathophysiological link.

### 3.4 Development of a simple risk-stratification tool

**Figure 3:**
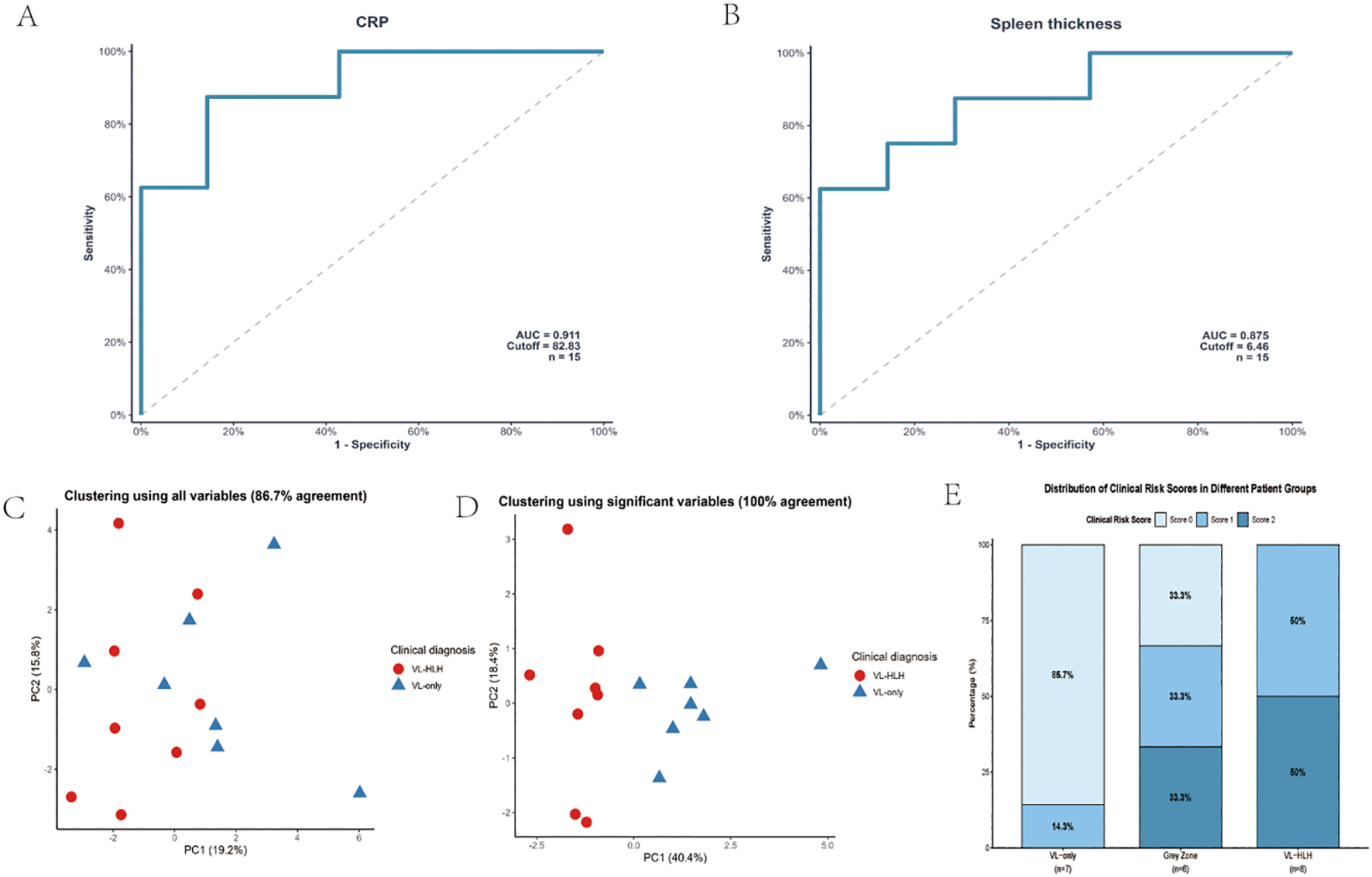
A risk stratification tool based on CRP and spleen thickness for visceral leishmaniasis and its discriminative performance. **(A)** Receiver operating characteristic curve of CRP for distinguishing VL-only from VL-HLH; **(B)** Receiver operating characteristic curve of spleen thickness for distinguishing VL-only from VL-HLH; **(C)** Unsupervised k-means clustering using all evaluated variables achieved 86.7% agreement with clinical classification; **(D)** Unsupervised k-means clustering using the nine significant variables (p < 0.05), including CRP and spleen thickness, achieved 100% perfect separation between VL-only and VL-HLH patients; **(E)** Distribution of the score (0–2 points) across different disease states.

Based on CRP and spleen thickness, which showed significant differences in baseline characteristics (**Table 1**), this study constructed a simple risk−stratification tool for discriminating between VL−only and VL−HLH. ROC analysis showed that CRP and spleen thickness had good discriminative ability, with an area under the curve (AUC) of 0.911 (95% CI: 0.714–1.000) and 0.875 (95% CI: 0.661–1.000), respectively (**Figures 3A, B**). To further validate the discriminatory power of these two parameters, unsupervised k-means clustering was performed on patients in the VL-only and VL-HLH groups. Using all evaluated variables, clustering achieved 86.7% agreement with clinical classification (**Figure 3C**). The Wilcoxon rank-sum test further identified nine variables with significant between-group differences (p < 0.05), including CRP and spleen thickness. Clustering using only these nine variables achieved perfect separation between VL-only and VL-HLH patients (100% agreement) (**Figure 3D**). Variable elimination analysis revealed that removing spleen thickness or HGB reduced the agreement rate from 100% to 87% (a 13% decrease), while removing CRP, PT, or D-dimer reduced it to 93% (a 7% decrease each) (**Supplementary Figure 1**). However, adding HGB to the CRP plus spleen thickness model did not improve performance; instead, the agreement rate decreased from 86.7% to 80.0%, indicating that HGB provided no additional discriminative value beyond the two-variable model (**Supplementary Figure 2**). Although PT and D-dimer also showed some impact in the elimination analysis, their AUC values were both 0.821, which were lower than those of CRP and spleen thickness (**Supplementary Figures 3**, **4**). Considering both discriminative performance and clinical accessibility, we selected CRP and spleen thickness to construct the scoring model. Considering data distribution and clinical practicality, we set the stratification thresholds at CRP > 80 mg/L and spleen thickness > 6.5 cm, thereby establishing a 0–2 point scoring system. To further validate the stability of the scoring system, we performed leave-one-out cross-validation (LOOCV) using a cutoff of ≥1 point. The original accuracy was 93.3% (sensitivity 100%, specificity 85.7%). LOOCV yielded an accuracy of 93.3%, identical to the original accuracy, indicating that the scoring system is stable and does not rely on any single subject. Among the 21 enrolled patients, this stratification revealed a clear discriminatory pattern. The majority (85.7%, 6/7) of patients in the VL-only group scored 0 points. Patients in the Grey Zone group were evenly distributed across scores of 0, 1, and 2. In contrast, all patients in the VL-HLH group scored either 1 or 2 points (50% each) (**Figure 3C**). The Jonckheere-Terpstra test for trend confirmed a statistically significant increasing trend in scores with greater disease severity (from VL-only, to Grey Zone, to VL-HLH) (P = 0.002), indicating that a higher score was associated with a greater likelihood of belonging to the VL-HLH group. In summary, the simple combination of CRP and spleen thickness can be effectively translated into an intuitive risk−stratification tool, demonstrating significant clinical utility for distinguishing disease severity and facilitating the early identification of patients at high risk of developing HLH.

## 4 Discussion

This retrospective cross-sectional study, analyzing 21 patients with VL, identified and characterized three distinct clinical profiles: VL-only, Grey Zone, and VL-HLH. The key finding indicates that, in the context of VL, the clinical phenotype associated with HLH does not represent a wholesale recapitulation of classic diagnostic criteria. Instead, it is more prominently characterized by a unique pathophysiological pattern centered on marked splenomegaly, accompanied by significant systemic inflammation and coagulopathy.

Our findings reveal a discrepancy between the clinical indicators predictive of disease progression in VL and the core laboratory parameters underpinning the conventional HLH-2004 diagnostic criteria. Specifically, compared to the VL-only group, the disease progression groups exhibited characteristic systemic inflammatory activation (represented by elevated CRP), coagulopathy (prolonged prothrombin time and elevated D-dimer), and marked splenomegaly. In contrast, ferritin and fibrinogen—core parameters of the diagnostic criteria—showed no statistically significant differences across the three groups. This discrepancy underscores the diagnostic complexity of VL-HLH. First, the discriminative value of ferritin, a cornerstone of the traditional criteria, was limited in our study population, which aligns with findings from a previous systematic review. That review explicitly noted that even ferritin levels >3,000μg/L, or exceeding 50,000μg/L, have become highly non-specific in critically ill adult patients (16). In the present study, the intense inflammatory drive inherent to VL infection itself was sufficient to provoke a pronounced elevation in ferritin, thereby diminishing its utility in distinguishing between isolated infection and VL-HLH. Secondly, triglycerides—another classical diagnostic marker for HLH—also did not show a significant increase in our study, a finding likely attributable to its susceptibility to various confounding factors and inherent limitations in diagnostic specificity and sensitivity. In contrast, the behavior of the third core parameter, fibrinogen (FIB), presents a more insightful pathophysiological puzzle. In the VL-HLH group, despite clear evidence of coagulation factor consumption (prolonged PT) and hyperfibrinolysis (elevated D−dimer), FIB levels did not exhibit the anticipated consumptive decrease. The underlying mechanism may involve a dynamic equilibrium between two opposing forces: on one hand, HLH itself can trigger severe cytokine storm-mediated endothelial injury and activation of the coagulation and fibrinolytic systems, a classic process that typically leads to consumptive hypofibrinogenemia (17); on the other hand, the intense and sustained inflammatory drive of visceral leishmaniasis may potently stimulate compensatory hepatic fibrinogen synthesis, likely mediated by cytokines such as IL−6 (18). Consequently, the observed FIB level represents the net effect of consumption versus compensatory synthesis. Our data suggest that compensatory synthesis may predominate at certain stages, which explains why not all patients with infection-associated HLH develop hypofibrinogenemia (19). Therefore, directly applying the HLH-2004 criteria to infection-associated HLH, particularly in visceral leishmaniasis, is problematic.

To gain a deeper understanding of the coordinated changes among key indicators during disease progression, we performed correlation analysis. The results revealed that multiple core indicators did not vary in isolation but formed a tightly interconnected network, providing systemic-level evidence for the widespread pathological damage in VL. Dimer and lactate dehydrogenase (LDH) showed the strongest positive correlation within this network(r = 0.74), and both were significantly correlated with prothrombin time (PT). This pattern directly indicates that coagulation activation and tissue-cellular damage are highly synchronized in visceral leishmaniasis. Their concurrent elevation collectively points to a more severe systemic pathophysiological state driven by hyperinflammation. This finding aligns with the classic pathological model of HLH, in which a dysregulated cytokine storm concurrently drives both coagulopathy and organ damage (20, 21).The significant positive correlation between ferritin and D-dimer (r=0.63) delineates a specific link between inflammation and coagulation. Serum ferritin is considered a useful biomarker that reflects the extent of macrophage activation (22). Their synchronous increase suggests that more intense macrophage activation is associated with stronger activation of the coagulation system. This correlation is consistent with the known mechanism of “immunothrombosis,” whereby activated macrophages can directly initiate the extrinsic coagulation pathway by releasing procoagulant substances such as tissue factor (23, 24). Therefore, we hypothesize that in VL, macrophages infected by the pathogen constitute the central nexus connecting inflammation and coagulation. This study found consistent positive correlations among white blood cell count (WBC), HGB, and platelet count (PLT), with a particularly strong correlation observed between WBC and HGB (r = 0.68). This coordinated pattern aligns with the established multifactorial mechanisms of pancytopenia in VL—including hypersplenism, bone marrow suppression, immune destruction, direct parasitic infection, and chronic inflammation (25)—suggesting that these pathological processes exert a synchronous and directionally similar global impact on granulocytic, erythroid, and megakaryocytic hematopoiesis. Interestingly, ferritin showed a positive correlation with hemoglobin (r = 0.53), which contrasts with the negative correlation typically expected in classic HLH theory. This discrepancy may be explained by the following: under conditions of severe infection, the specificity of ferritin as a diagnostic marker for HLH is significantly diminished (16). Its elevated levels primarily reflect the systemic inflammatory burden rather than specifically quantifying the degree of bone marrow suppression. Simultaneously, hemoglobin levels are regulated by multiple factors, including bone marrow suppression, hemolysis, blood loss, and compensatory responses to hypoxia (26). Therefore, the observed statistical association between ferritin and hemoglobin is more likely driven by a shared, intense systemic inflammatory background and influenced by other confounding factors, rather than indicating a straightforward causal relationship of suppression. The strong positive correlation between triglyceride (TG) and lactate dehydrogenase (LDH) (r=0.63) likely reflects concurrent lipid metabolism disturbance and cellular damage in a state of active macrophage phagocytosis (27).A negative correlation was observed between age and LDH (r = -0.54), suggesting that younger patients in this study population had higher LDH levels, a biomarker commonly associated with tissue damage. This observation could be related to age-related differences in immune response dynamics, although further studies are needed to confirm this finding.

More importantly, PET/CT metabolic imaging localized these systemic disturbances to a key target organ—the spleen. Given that the spleen is one of the core target organs parasitized by *leishmania* (28, 29), our data show that the spleen was the most consistently and metabolically active involved organ in visceral leishmaniasis (9/9, 100%). Splenic metabolic activity (SUVmax) showed a very strong positive correlation with D-dimer levels (r=0.82), reflecting systemic coagulation activation, and also correlated significantly with ferritin levels (r=0.69), an indicator of macrophage activation/inflammation. From a pathological perspective, its characteristic pathological change—massive accumulation of parasite-laden macrophages—forms the cytological basis for its high local metabolic activity (30). During infection and inflammation, activated monocytes/macrophages significantly increase local fluorodeoxyglucose uptake through mechanisms such as upregulation of glucose transporters (31). Previous studies have demonstrated that serum TGF-β levels are significantly elevated in patients with VL (32, 33). Gauthier et al. (34) further elucidated the molecular mechanism by which TGF-β regulates macrophage metabolism: TGF-β upregulates the glycolytic enzyme PFKL through the mTOR-c-MYC pathway, thereby enhancing macrophage glycolysis—a metabolic activity reflected by FDG-PET SUVmax. Notably, this study also found that TGF-β-induced enhancement of macrophage glycolysis is associated with coagulation dysfunction. This observation, together with our finding of a strong positive correlation between splenic SUVmax and D-dimer (r = 0.82), suggests a potential link between macrophage glycolytic metabolism and coagulation activation. Together, these findings support the notion that metabolic reprogramming of activated macrophages within the spleen may be involved in the regulation of systemic inflammation-coagulation networks. Furthermore, *Leishmania* infection is known to disrupt host iron homeostasis. Banerjee and Datta demonstrated that *Leishmania* infection promotes iron accumulation within macrophages by upregulating transferrin receptor 1 (TfR1) to enhance iron uptake and downregulating the iron exporter Nramp1 to trap iron inside cells (35). *Leishmania* parasites are heavily dependent on efficient iron acquisition from the host iron pool for survival and virulence (35). Because iron is retained within macrophages and cannot be released into the bloodstream, systemic available iron is reduced, leading to anemia; the spleen subsequently enlarges as a compensatory site for extramedullary erythropoiesis. Ferritin is a well-established marker of intracellular iron storage. Therefore, the positive correlation between splenic SUVmax and ferritin levels (r = 0.69) observed in our study may reflect increased iron storage in activated splenic macrophages. It should be noted that the spleen, as a major site of erythrocyte recycling, does exhibit baseline physiological metabolic activity. However, in our study, all nine patients demonstrated markedly elevated splenic SUVmax (range 4.44-9.85) with unequivocal diffuse hypermetabolism. This degree of elevation far exceeds the physiological range. Therefore, the extremely high splenic SUVmax observed in this study largely reflects pathological immune activation of splenic macrophages rather than normal physiological activity. Combined with the previously discussed role of macrophages in driving coagulation and their function as key drivers of hyperinflammation in VL (23, 24, 36), our data strongly suggest that the spleen is not merely a central site of anti-parasite immune response; the activated macrophages within it may act as a functional hub driving the systemic “inflammation-coagulation” vicious cycle. In this study, we also measured serum IL-6 levels in the same nine patients who underwent PET-CT. The median IL-6 levels in the VL-only, Grey Zone, and VL-HLH groups were 35.44 pg/mL, 221.30 pg/mL, and 23.30 pg/mL, respectively, with no clear increasing trend across disease severity groups, and substantial inter-individual variability was noted in the VL-HLH group (range 1.05-167.40 pg/mL). This finding may be explained by the kinetic characteristics of IL-6. Waage et al. (37) demonstrated that IL-6, as a core cytokine of the acute-phase response, has an extremely short serum half-life (approximately 1–2 hours) and exhibits a burst-release pattern—rapid peaking followed by swift clearance. Therefore, a single time-point measurement may not accurately capture the true IL-6 burden, especially when blood sampling is not synchronized with the peak of the cytokine storm, potentially masking the association between IL-6 levels and disease severity. Despite the absence of a clear trend, the Grey Zone patient exhibited a markedly elevated IL-6 level (221.30 pg/mL), comparable to the highest value in the VL-HLH group (167.40 pg/mL), and also showed the highest splenic SUVmax (9.85), suggesting an inflammatory and metabolic phenotype more closely aligned with the VL-HLH group. Thus, while a single time-point IL-6 measurement may not serve as an independent indicator of disease severity, extreme elevations still carry important clinical implications.

To address challenges such as diagnostic delay when applying the HLH−2004 criteria in resource−limited settings, we developed a simple risk−stratification tool based on readily accessible indicators (CRP and spleen thickness). This study demonstrated that the model exhibited clear discriminative potential, effectively distinguishing uncomplicated infection from HLH and enabling risk stratification for patients in the Grey Zone. Unsupervised k-means clustering analysis further validated that CRP and spleen thickness are the core discriminative parameters of this tool. The tool’s primary strength lies in its exceptional clinical accessibility and immediacy. The assessment relies entirely on routine laboratory testing and bedside imaging, without the need for complex, time−consuming specialized immunological assays. This feature allows it to be directly translated into a practical decision−support tool for primary care or endemic settings, providing front-line clinicians with an intuitive decision-support tool for early risk stratification.

Several limitations of this study should be acknowledged. First, as a single-center retrospective study with a relatively limited sample size (n = 21), the statistical power to detect differences in certain parameters was limited. Consequently, we were unable to adjust for potential confounders (e.g., age, baseline liver/kidney function) using multivariable regression analysis, nor could we derive the risk assessment tool from rigorous multivariable modeling. Instead, the tool was directly constructed based on two indicators (CRP and spleen thickness) selected through univariate analysis. Therefore, this tool remains exploratory in nature and warrants further validation in subsequent studies. Second, as a cross-sectional study, it lacks longitudinal follow-up data, which limits its ability to assess the predictive performance of this tool over time; prospective longitudinal studies are needed for further validation. Third, this study focused specifically on patients with VL, and a pure HLH (non-VL-related) control group was not included. Pure HLH itself comprises a heterogeneous group of etiologies (e.g., lymphoma, EBV, other infections, autoimmune diseases), and a simple comparison with such a mixed group could be confounded by underlying cause. Therefore, whether the clinical and imaging features observed in VL-HLH are specific to this condition or shared with broader HLH remains to be investigated in well-designed studies with etiologically stratified control groups. Fourth, due to resource limitations, key functional immunological assays—including soluble CD25 (sCD25) and NK cell activity—were not available for all patients, which restricted a comprehensive characterization of the immunological phenotype. Fifth, the PET/CT metabolic imaging analysis was based on a small subgroup (n = 9); although the “splenic metabolic hub” hypothesis generated from these data is mechanistically intriguing, it requires validation in larger cohorts. Additionally, due to the retrospective design, the exact date of fever onset was based on patient recall and may have had recall bias, which precluded precise calculation of the time interval between fever onset and PET/CT examination. Sixth, although most patients had no pre-hospital corticosteroid exposure, a small number (n=4) had received corticosteroids before admission, which may have influenced baseline inflammatory markers; however, given the small proportion, the impact is likely limited. Future studies should systematically document pre-hospital medication use, including the indication, type, dose, and duration of corticosteroids, to better assess and adjust for their potential impact. Additionally, elevated C-reactive protein (CRP) is not specific to VL-HLH, and concurrent infections could not be completely excluded, though routine screening did not identify other definite infectious etiologies in most patients. Future prospective studies with larger sample sizes should include a control group of patients with other febrile illnesses to further validate the specificity of CRP for distinguishing VL-HLH from other causes of fever and inflammation.

Nevertheless, this study provides important foundational data and preliminary evidence from a relatively rare clinical sample. To address these limitations, future research should focus on the following directions. First, large-scale, multicenter prospective cohort studies are warranted to externally validate and optimize the risk stratification tool developed in this study, while exploring the incorporation of dynamic changes in coagulation and inflammatory markers into the model to enhance its predictive timeliness and accuracy. Second, future studies with etiologically stratified pure HLH (non-VL-related) control groups are needed to determine whether the clinical and imaging features observed in this study are specific to VL-HLH or shared across different HLH etiologies, thereby further validating the specificity of our scoring system. Third, in-depth mechanistic investigations employing multi-omics technologies are needed to systematically elucidate the molecular networks through which splenic macrophage activation drives immunothrombosis and cytokine storms in the context of visceral leishmaniasis. Fourth, the potential value of metabolic imaging parameters—such as those derived from PET/CT—in treatment monitoring and prognostic assessment warrants further investigation.

In conclusion, this study reveals a pathophysiological model centered on the “inflammation-coagulation-spleen” axis and establishes a simple risk stratification tool to assist in risk stratification for VL patients at hospital admission to identify those at high risk of HLH. This work provides a new perspective for understanding this severe complication and lays a preliminary foundation for early recognition and stratified management in resource-limited settings.

## Data Availability

The raw data supporting the conclusions of this article will be made available by the authors, without undue reservation.
