# Supplementary material for: Clinical and PET/CT metabolic imaging characteristics across the evolving spectrum of visceral leishmaniasis and associated hemophagocytic lymphohistiocytosis
Source: Front Immunol. 2026 Jul 13;17:1812725. doi: 10.3389/fimmu.2026.1812725 (PMC13402202; doi:10.3389/fimmu.2026.1812725)

**Figure S1. Variable elimination analysis.** Removing spleen thickness or HGB reduced the clustering agreement rate from 100% to 87% (a 13% decrease), while removing CRP, PT, or D-dimer reduced it to 93% (a 7% decrease each).


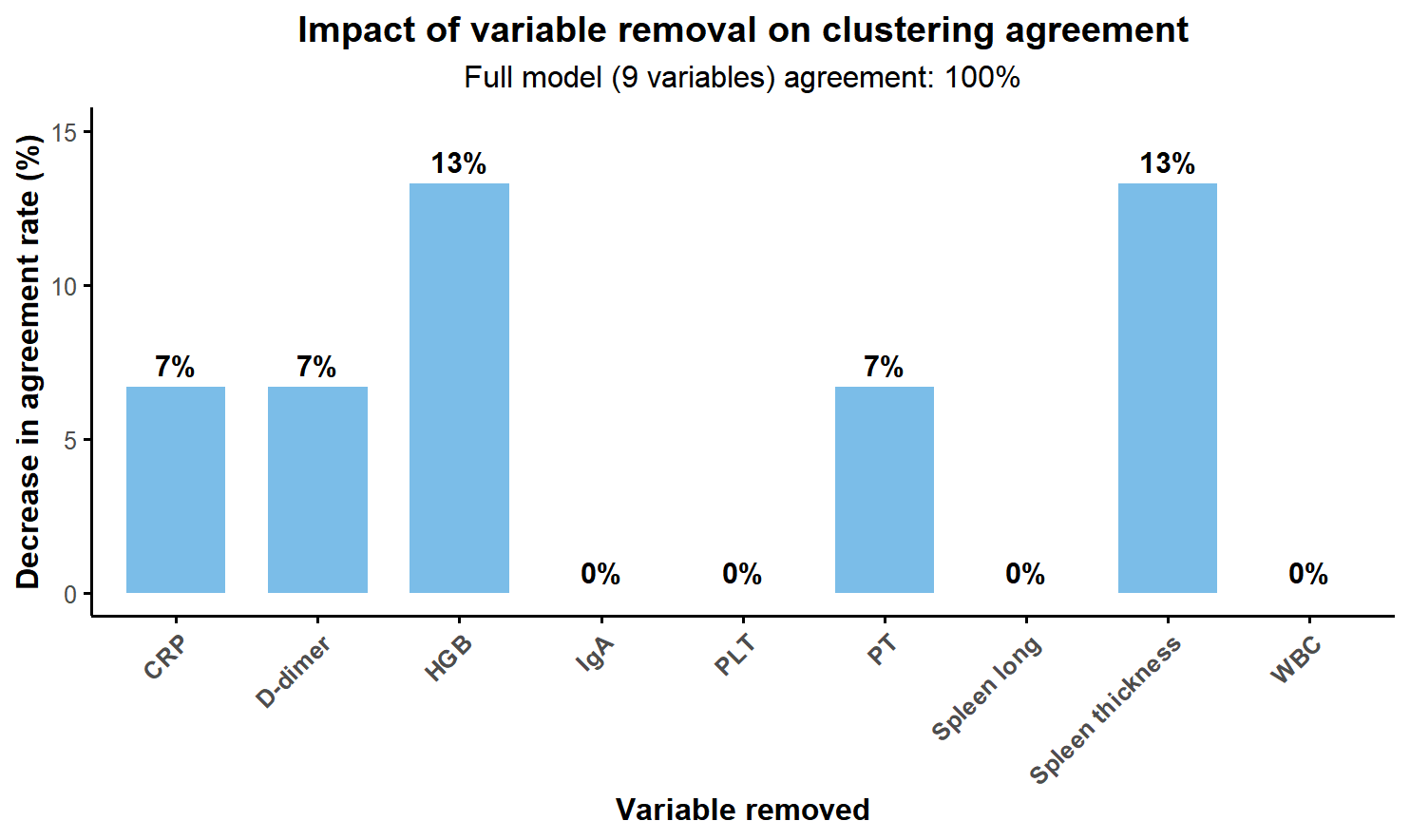


**Figure S2. Adding HGB to the CRP plus spleen thickness model.** The agreement rate decreased from 86.7% to 80.0%, indicating that HGB provided no additional discriminative value beyond the two-variable model.


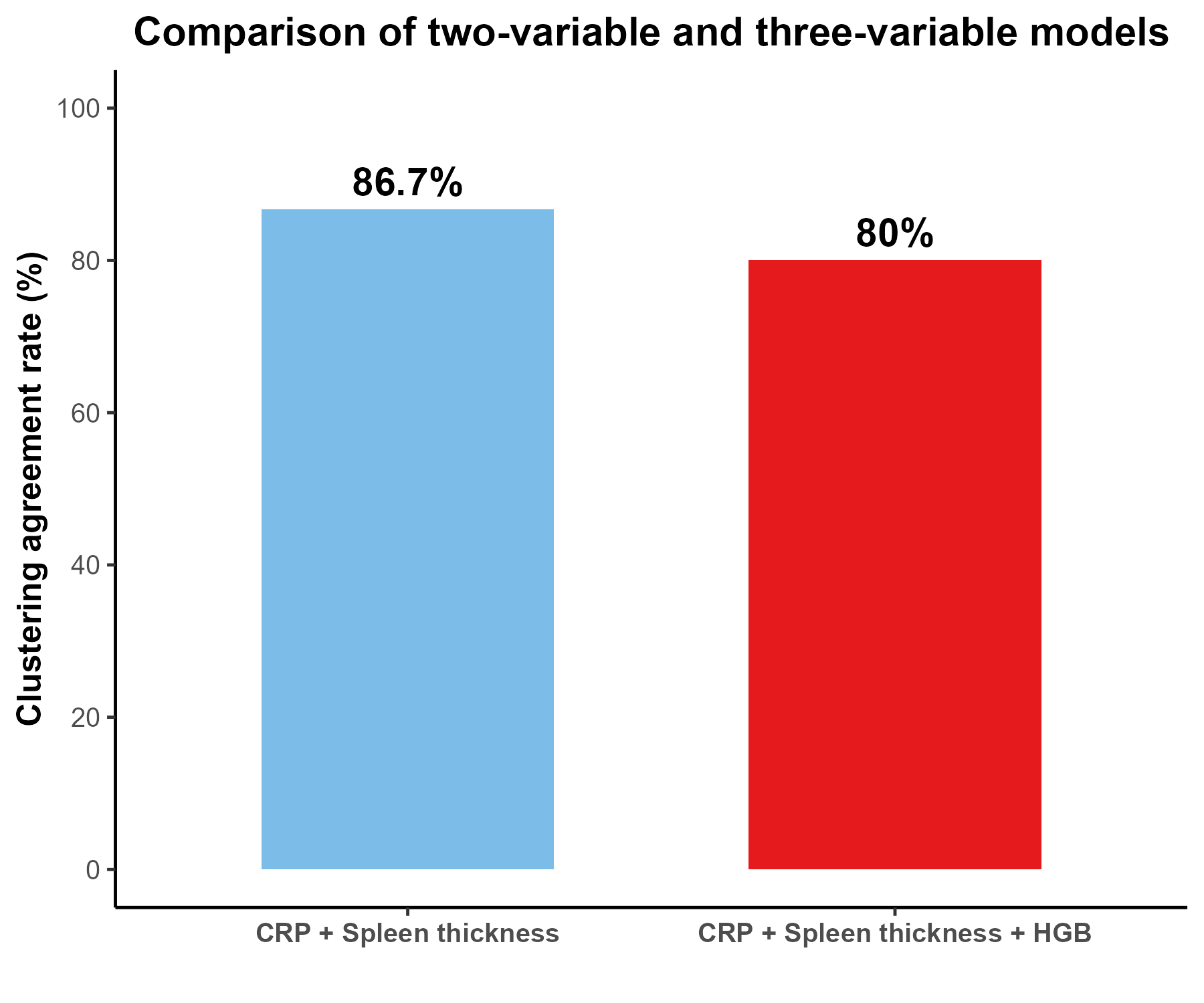


**Figure S3. Receiver operating characteristic curve of PT for distinguishing VL-only from VL-HLH (AUC = 0.821).**


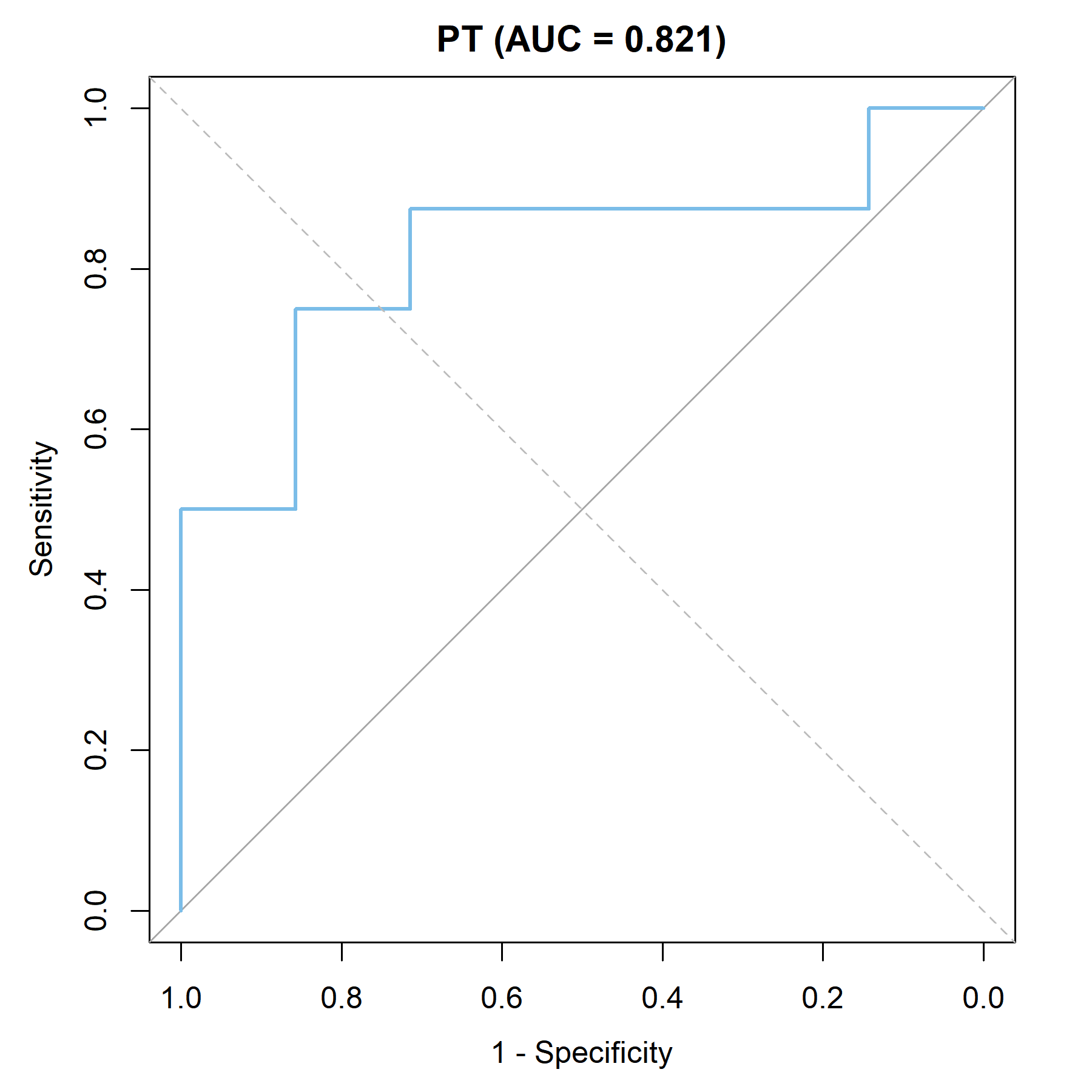


**Figure S4. Receiver operating characteristic curve of D-dimer for distinguishing VL-only from VL-HLH (AUC = 0.821).**


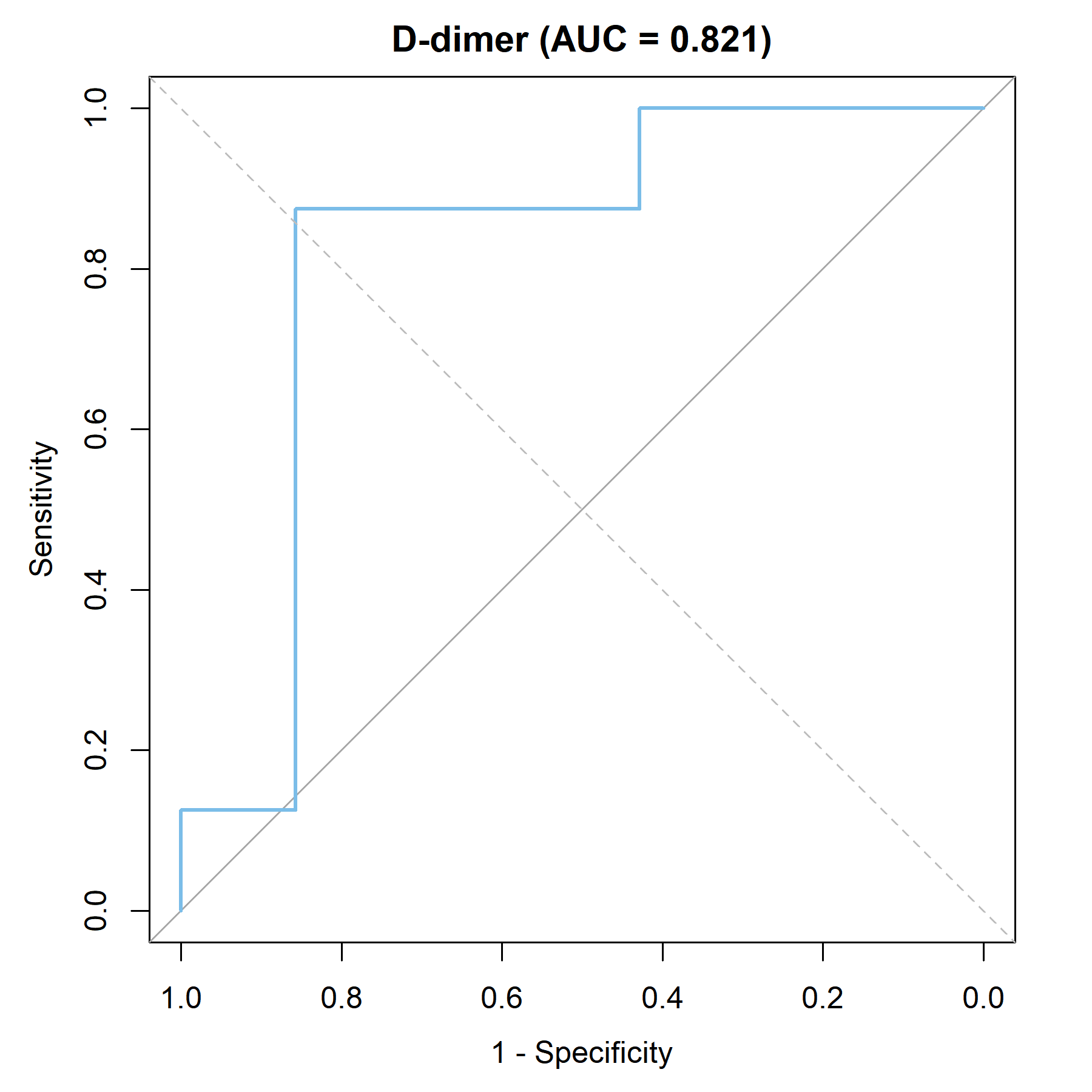

Supplement: Supplementary file 1 [file DataSheet1.doc]
