# Supplementary material for: Clinical and PET/CT metabolic imaging characteristics across the evolving spectrum of visceral leishmaniasis and associated hemophagocytic lymphohistiocytosis
Source: Front Immunol. 2026 Jul 13;17:1812725. doi: 10.3389/fimmu.2026.1812725 (PMC13402202; doi:10.3389/fimmu.2026.1812725)
Supplement: Supplementary file 2 [file Table1.doc]

Supplementary Table 1: Individual HLH-2004 laboratory indicators of six patients in the Grey Zone

| ID | Fever | Splenomegaly | Ferritin ≥500 ng/L | Cytopenia (≥2 lines) | Hemophagocytosis | HyperTG/ HypoFIB | NK activity | Elevated sCD25 | Number of criteria met |
| --- | --- | --- | --- | --- | --- | --- | --- | --- | --- |
| 01 | √ | √ | √ | × | × | × | / | / | 3 |
| 02 | × | √ | √ | √ | × | × | / | / | 3 |
| 03 | √ | √ | √ | √ | × | × | / | / | 4 |
| 04 | √ | √ | √ | × | × | × | / | √ | 4 |
| 05 | √ | √ | √ | × | × | × | / | √ | 4 |
| 06 | √ | × | √ | √ | × | √ | / | / | 4 |

Note:√, criterion met; ×, criterion not met; /, not tested; HyperTG/HypoFIB, hypertriglyceridemia and/or hypofibrinogenemia; NK activity, natural killer cell activity.
